# Clinical Success Rate of Compomer and Amalgam Class II Restorations in First Primary Molars: A Two-year Study

**DOI:** 10.15171/joddd.2015.018

**Published:** 2015-06-10

**Authors:** Faezeh Ghaderi, Ali Mardani

**Affiliations:** ^1^Associate Professor, Department of Pediatrics dentistry, Shiraz University of Medical Sciences, Shiraz, Iran; ^2^Associate Professor, Department of Pediatrics dentistry, Shiraz University of Medical Sciences, Shiraz, Iran

**Keywords:** Amalgam, compomer, dental restorations, primary teeth

## Abstract

***Background and aims.*** The majority of failures in Class II amalgam restorations occur in the first primary molar teeth; in addition, use of compomer instead of amalgam for primary molar teeth restorations is a matter of concern. The aim ofthe present study was to compare the success rate of Class II compomer and amalgam restorations in the first primary molars.

***Materials and methods.*** A total of 17 amalgams and 17 compomer restorations were placed in 17 children based on a split-mouth design. Restorations were assessed at 12- and 24-month intervals for marginal integrity, the anatomic form and recurrent caries. Data were analyzed with SPSS 11. Chi-squared test was applied for the analysis. Statistical significance was set at P<0.05.

***Results.*** A total 34 restorations of 28 restorations (14 pairs) of the total restorations still survived after 24 months. Compomerrestorations showed significantly better results in marginal integrity. Recurrent caries was significantly lower incompomer restorations compared to amalgam restorations. Cumulative success rate at 24-month interval was significantlyhigher in compomer restorations compared to amalgam restorations. There was no statistically significant difference inanatomic form between the two materials.

***Conclusion.*** Compomer appears to be a suitable alternative to amalgam for Class II restorations in the first primary mo-lars.

## Introduction

The increasing demand for esthetic restorations and the concern over harmful effect of amalgam because of its mercury content are some of the main reasons for limitation of its use in restoring primary teeth.^1-3^ However, there is still controversy regarding the selection of the optimal restorative material.^4^ Some tooth-colored restorative materials are now available, including glass-ionomers (GI), resin-modified glass-ionomers (RMGI) and compomer as alternatives to amalgam.^5^ However, low flexural and compressive strengths and wear resistance of GI have resulted in less survival rates compared to amalgam in primary molars.^6^ The application of composite resins even with simplified adhesive systems is a very technique-sensitive procedure, especially when an absolutely dry operation filed cannot be guaranteed.^7^Compomers have been widely accepted by practitioners because of ease of handling and improved physical and chemical properties.^8^ In addition, compomers release fluoride ions so they might have caries protection effects.^9^However, there is some controversy in the caries preventive effect of compomer because of the amount of fluoride released.^9^ From longevity point of view, the greatest longevity was found for class I composite restorations and the lowest for class II amalgam restorations.^10^The most common failures in Class II amalgam restorations have been observed in the first primary molars.^11^ To the best of our knowledge, there is no research comparing the success rate of compomer and amalgam restorations only in the first molar teeth; therefore, the current study was designed to evaluate this issue. To shorten the follow-up period, we performed the study on children and teeth with poor prognosis from restoration longevity point of view. Therefore, the current study was performed on class II cavities in first primary molars and children with poor oral hygiene and low socioeconomic background. The clinical success rate of compomer and amalgam restorations on first primary molars was evaluated after one and two years of follow-up periods.

## Materials and Methods

A total of 17 healthy children (ASA I) from urban areas of Faroogh participitated in this study. All the 6‒8-year-old children who participated in the study had low socioeconomic status. Their oral health was poor as evidenced by high dental plaque accumulation and dental caries. None of the subjects reported a history of dental visit while they had several dental caries due to low socioeconomic status. Subjects with paired minimal class II cavities of first primary molars were included in the study.

Periapical radiography of the first primary molars was recommended to make sure that the dental caries had not resulted in pulp involvement.

### Exclusion Criteria

Children with behavioral problems, with primary first molars needing pulp therapy (the teeth with a history of pain, fistula, mobility, periapical radiolucency, furcation involvement, widening of periodontal ligament) and with primary first molars without an adjacent tooth were excluded from the study

The clinical procedures of the study were explained and consent forms were obtained from parents. The Committee of Research and Ethics of Shiraz University of Medical Sciences approved all the aspects and steps of the protocol.

A split-mouth design with an identical pair of minimal class II cavities of matched tooth types in same dental arch was used.

Primary first molars in each subject were randomly restored with compomer or amalgam.

For amalgam restorations a conventional cavity design was prepared according to Black's principle. For compomer restorations, conservative cavities were prepared, although the retention‏ for compomer was supplied by an adhesive material. Matrix bands and wedges were used to place both materials in cavities. A conventional amalgam mix was used (SINA, IRAN). For compomer (Compomer F2000, 3M, USA) restorations, a self-etching adhesive (Adper Easy Bond One Bottle, 3M ESPE, USA) was applied according to the manufacturer's instructions and cured for 40 seconds.

Both restorations were evaluated after 12, 24 months by clinical and radiographic examinations. A dental probe (Shengkang Co., Ltd, China) was used for the assessment of marginal integrity. If the probe just caught it and did not fall in it was considered M1, and if the explorer tip fell in but the dentin was not exposed it was scored M2. When the dentin was visible it was scored M3. In the M4 score, the dental restoration was lost or fractured as shown in Table 1.

**Table 1:** The assessment criteria used to evaluate restorations

**Grade**	**Approximal and occlusal marginal integrity**
M1	Restoration adapts closely to tooth along margins.
M2	Probe catches in the marginal gap; dentin not visible.
M3	Probe catches in the gap; dentin visible; restoration has failed.
M4	Restoration is fractured or lost; restoration has failed.
**Grade**	**Anatomic form**
**Grade**	**Anatomic form**
A1	Restoration is continuous with the anatomy of tooth.
A2	Restoration material lost but no dentin exposed.
A3	Dentin exposed by loss of material; restoration has failed.
**Grade**	**Recurrent caries**
R1	Absent
R2	Present; restoration has failed.

Recurrent carries was assessed by a bitewing radiograph.

Then the score was determined based on criteria shown in Table 1. The modified United States Public Health Service (USPHS) criteria were used by Kilpatrik et al (1995) (Table 1).^12^

Investigators evaluated the restoration at follow-up visits according to the criteria used by modified USPHS (Kilpatrik et al) for marginal integrity (MI), anatomic form and recurrent carries as follows:

Marginal integrity was ranked as successful when it scored M1 or M2, similar to anatomic form (A1 or A2). A restoration was considered to be successful when it scored marginal integrity (MI) and anatomic form (AF) grade of either 1 or 2, with no evidence of recurrent carries (cumulative success rate).

Out of 34 restorations, 28 were assessed at the 24-month recall.

Data were analyzed by SPSS 11. Chi-squared test was applied for analysis. Statistical significance was defined at P < 0.05.

## Results

After 12 months all the patients returned for the recall visit. At 24 months three patients (6 restorations) did not return for the visit. Recall rates for patients at 12 months and 24 months were 100% and 82%, respectively. Statistical analysis showed no significant differences between the two groups in marginal integrity (P = 0.554), anatomic form (P = 0.554), recurrent carries (P = 0.235) and cumulative successes rates (P = 0.324) after 12 months (Table 2).

**Table 2:** Clinical success rates of compomer and amalgam in marginal integrity, anatomic form, recurrent carries and cumulative success rates at 12-month recall

**Clinical evaluation parameter**	**Success rates in amalgam restorations**		**Success rate in Compomer restorations**		
	**N**	**Percent**	**N**	**Percent**	**P-value**
**Marginal integrity**	28	90.3%	27	87.1%	P=0.554
**Anatomic form**	27	87.1%	28	90.3%	P=0.554
**Recurrent carries**	4	12.9%	2	6.5%	P=0.235
**Cumulative success**	24	77.4%	27	87.1%	P=0.324

Compomer restorations showed significantly better performance in marginal integrity (P = 0.043), recurrent carries (P = 0.003) and cumulative successes rates (P = 0.021) in comparison with amalgam restorations after 24 months (Table 3).

**Table 3:** Clinical success rates of compomer and amal-gam in marginal integrity, anatomic form, recurrent carries and cumulative success rates at 24-month recall

**Clinical evaluation parameter**	**Success rate in Amalgam Restorations**		**Success rate in Compomer restorations**		
	**N**	**Percent**	**N**	**Percent**	**P-value**
**Marginal Integrity**	20	71.4%	24	85.7%	P=0.043
**Anatomic Form**	22	78%	24	85%	P=0.432
Recurrent Carries	9	25%	3	10%	P=0.003
**Cumulative success**	19	67.9%	24	85.7%	P=0.021

There were no significant differences between the two groups in anatomic form after 24 months (P = 0.432; Table 1).

## Discussion

The results of the current study showed no statistically significant differences between amalgam and compomer restorations in marginal integrity, anatomic form and recurrent carries at 12-month follow-up period.

There was a statistically significant difference in marginal integrity between amalgam and compomer restorations at 24-month recall visit. The superiority of compomer restoration in marginal integrity might be attributed to its adhesive property, while amalgam relies purely on mechanical retention.^13^

Our finding is consistent with Duggal et al,^13^ who reported that the compomer restoration material had better marginal integrity in comparison with amalgam in primary molar restorations. The results showed that anatomic form of compomer was better than amalgam after 24 months; however, the difference was not statistically significant. Although compomer abraded from the occlusal surface, replacement of restorations due to its surface wear was minimal.

The pulp chamber of first primary molar, especially on the mesial side, is very close to the tooth surface, making it difficult to provide adequate retention for an amalgam restoration.^14^ Meanwhile, considerable amounts of amalgam restorations were fractured or totally lost.

There were statistically significant differences in the development of dental carries between amalgam and compomer after 24 months. Dental caries usually occurs because of marginal micro-gaps.^15^In the current study 20% of amalgam restorations were totally lost, all of which showed recurrent carries. Kavvadia et al^16^ reported that dental caries occurred at a rate of 3‒9% around amalgam restorations in comparison with 5‒6% in compomer restorations.In this study recurrent carries in amalgam restorations occurred at a high rate (25%), which might be attributed to the high rate of amalgam loss and poor oral hygiene of the children.

The results of the present study showed that clinical cumulative success of compomer in Class II restorations in the first primary molars was significantly higher than the amalgam restorations at 24-month follow-up. This is not consistent with Kavvadia et al^16^and Soncini et al,^17^ who showed superior performance of amalgam restorations in comparison with composer resins in posterior teeth. As it has been reported that amalgam restorations placed in the first primary molars have shorter survival times in comparison with those in the second primary molars,^11-14^the discrepancy may be contributed to the different teeth evaluated in different studies. We included only first primary molars needing Class II restorations, which is different from other similar studies considering Class I and II restorations in primary posterior teeth or permanent and primary posterior teeth.^16,17^

## Conclusion

Compomer showed higher success rates in Class II primary first molar restorations in comparison with amalgam. The clinical performance of compomer in primary first molars was more acceptable after two years compared to amalgam.

## Acknowledgments

The authors would like to acknowledge Research Deputy of Shiraz Dental School. The authors also would like to appreciate the participation of the children in the study. The manuscript was prepared from an undergraduate dissertation for a doctorate degree in dentistry at Shiraz University of Medical Sciences (No. 1391).
